# An Unusual Case of Chronic Cough: Is It Beyond Simple Gastroesophageal Reflux Disease?

**DOI:** 10.7759/cureus.82449

**Published:** 2025-04-17

**Authors:** Grant Hughes, Austin Reynolds, Knogwan Yuenyongsagul, Samina Ayub, Abdul Waheed

**Affiliations:** 1 Family Medicine, Dignity Health East Valley, Gilbert, USA; 2 Family Medicine, Creighton University School of Medicine, St. Joseph's Hospital and Medical Center, Phoenix, USA; 3 Nursing, College of Nursing, University of Phoenix, Phoenix, USA; 4 Family Medicine, Dignity Health Medical Group Arizona, Chandler, USA; 5 Family Medicine, Dignity Health, Phoenix, USA; 6 Family and Community Medicine, Creighton University School of Medicine, Phoenix, USA; 7 Family Medicine, Dignity Health Medical Group Arizona, Gilbert, USA

**Keywords:** adenocarcinoma, cancer, chronic cough, esophageal carcinoma, gastroesophageal reflux disease, gerd

## Abstract

Esophageal carcinoma commonly presents with dysphagia, weight loss, or retrosternal pain. Less commonly, chronic cough can be the initial presenting symptom, posing a challenge to the physician making the diagnosis. More common etiologies of chronic cough may present similar to those of esophageal carcinoma, making it hard to distinguish malignancy from common causes of cough, such as upper airway cough syndrome (postnasal drip), asthma, and gastroesophageal reflux disease prior to proper imaging. In this case, a 77-year-old male with a one-year history of chronic cough was referred to an allergist and diagnosed with allergic rhinitis. One year later, the patient developed esophageal reflux symptoms and chest pain radiating to the back and visited the Cardiology as well as the Emergency Department. The patient was discharged and referred to gastroenterology, where an endoscopy with biopsy revealed grade 3 adenocarcinoma of the distal esophagus without extension into the cardia. He was treated with chemoradiation followed by distal esophagectomy and has been in remission since treatment for four years. This case displays the importance of keeping differentials broad when treatments for common diagnoses fail to deliver the results physicians would expect.

## Introduction

Esophageal cancer is a serious and often aggressive disease that originates in the tissue lining of the esophagus. Histologically, there are two main subtypes: squamous cell carcinoma and adenocarcinoma. Although squamous cell carcinoma is more common worldwide, the rates of esophageal adenocarcinoma in the United States continue to rise and have surpassed the incidence of squamous cell carcinoma [1]. Esophageal adenocarcinoma is a highly lethal malignancy with a poor prognosis, as the five-year survival rate is less than 20% [2].

Esophageal carcinoma arises from chronic inflammation and damage of the esophageal lining. This irritation can lead to the replacement of squamous epithelium with metaplastic columnar epithelium - a condition known as Barrett’s esophagus. Patients with long-standing and severe gastroesophageal reflux disease are most likely to develop Barrett’s esophagus, as chronic exposure to gastric acid leads to the disruption of the esophageal lining. Patients with Barrett’s esophagus have a 30- to 125-fold increased risk of developing esophageal adenocarcinoma. However, the absolute risk for developing esophageal adenocarcinoma in patients with Barrett’s esophagus remains low at 0.1-0.5% per year [3]. Although gastroesophageal reflux disease is a significant risk factor for esophageal carcinoma, one study showed a substantial minority (36%) of patients with esophageal carcinoma did not have preexisting gastroesophageal reflux disease [4]. With a notable portion of patients with esophageal adenocarcinoma presenting without gastroesophageal reflux disease symptoms, a deeper understanding of risk factors and screening strategies are needed for the early detection of esophageal adenocarcinoma.

Dysphagia is the most common presenting symptom in patients with esophageal carcinoma, but the presence of dysphagia indicates that the disease has already advanced to a later stage. Most patients with esophageal carcinoma who develop dysphagia already have T3-T4 disease, showing that identifying esophageal carcinoma before dysphagia symptoms occur is critical to improving survival rates [5].

Chronic cough is not typically thought of as a standalone presenting symptom of esophageal carcinoma. However, one study showed that laryngopharyngeal reflux symptoms (chronic cough, asthma, aspiration, hoarseness, globus, sore throat, and sinusitis) are more prevalent than the most typical gastroesophageal reflux disease symptoms (heartburn and regurgitation) in patients with esophageal carcinoma. This study showed that 54% of patients with esophageal carcinoma had laryngopharyngeal reflux symptoms, while 43% had typical gastroesophageal reflux disease symptoms. Overall, it was found that chronic cough was an independent risk factor for esophageal carcinoma [6]. Although early symptoms of esophageal carcinoma are vague, further evaluation of chronic cough, especially in conjunction with typical gastroesophageal reflux disease symptoms or other risk factors, may prompt earlier detection of esophageal carcinoma. This report presents the case of a patient with a chief complaint of chronic cough and how complex care in two distinct geographical regions along with a high persistent index of suspicion by the primary care physician led to a diagnosis of esophageal adenocarcinoma.

## Case presentation

A 77-year-old male presented to a Primary Care Physician in Arizona to establish care on February 11, 2019, with a chief concern of chronic cough for one year. He typically spends his winters in Arizona and the summers in Utah. He had a past medical history significant for coronary artery disease, hypertension, hyperlipidemia, and diabetes.

Previously in Utah (in 2018), the patient visited an otolaryngologist and was provided an antihistamine for a running diagnosis of allergic rhinitis secondary to a chief complaint of isolated chronic cough. One year later in Arizona (February 11, 2019), the patient described his cough as non-productive, with new accompanying symptoms of associated reflux, trouble swallowing, and intermittent shortness of breath. He went through a negative pulmonary work-up and was referred to a gastroenterology specialist, but he subsequently moved back to Utah for the summer before seeing the said specialist. When he returned to Arizona the next year, he made an appointment in January 2020, reporting worsening of his reflux with little alleviation from over-the-counter Tums. He also noted centralized chest pain that radiated to his back, prompting an in-office electrocardiogram. This electrocardiogram showed an incomplete right bundle branch block without available prior electrocardiogram for comparison. Because of his concurrent coronary artery disease, hypertension, and lipidemia, the patient was referred to a cardiologist in Arizona.

His reflux worsened before his appointment with cardiology, and he presented to the Emergency Department on February 27, 2020, where a cardiac workup was performed including an echocardiogram and stress test. His echocardiogram showed borderline concentric left ventricular hypertrophy and grade 1 diastolic dysfunction consistent with impaired ventricular relaxation but normal filling pressure. His ejection fraction was estimated to be 50% to 60%.

He was referred to gastroenterology for his continued reflux and underwent an esophagogastroduodenoscopy with biopsy on March 17, 2020. His esophagogastroduodenoscopy revealed a 3- to 4-cm mass with ulcerations in the distal esophagus just proximal to the gastroesophageal junction. Mild erythema was visualized in the antrum, which was compatible with gastritis. Pathology returned the same week with grade 3 adenocarcinoma of the distal esophagus without extension into the cardia. An endoscopic ultrasound completed in March 2020 revealed a "T3 N1 esophageal adenocarcinoma with mass extending into the muscularis propria and serosa as well as two 1 cm lymph nodes seen adjacent to the mass and two subcentimeter nodes seen proximal to the mass in the mediastinum." A positron emission tomography scan of the skull base to the thighs was also completed in March 2020, which did not show evidence of distant metastasis. The patient was subsequently treated with chemoradiation starting March 27, 2020, and followed by an Ivor Lewis esophagectomy with regional lymphadenectomy in June 2020.

**Figure 1:**
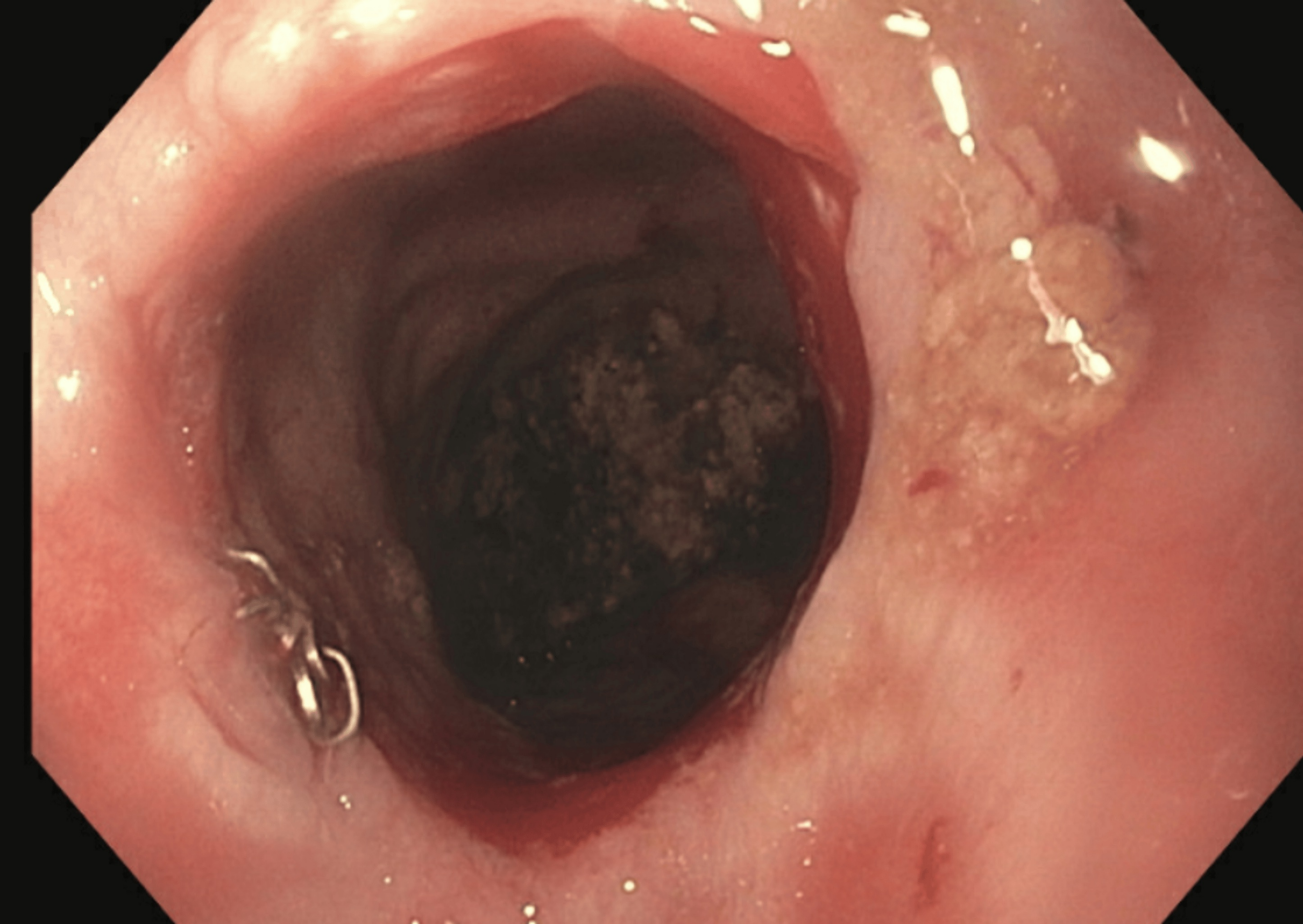
Esophagogastroduodenoscopy with dilation (November 11, 2020)

**Figure 2:**
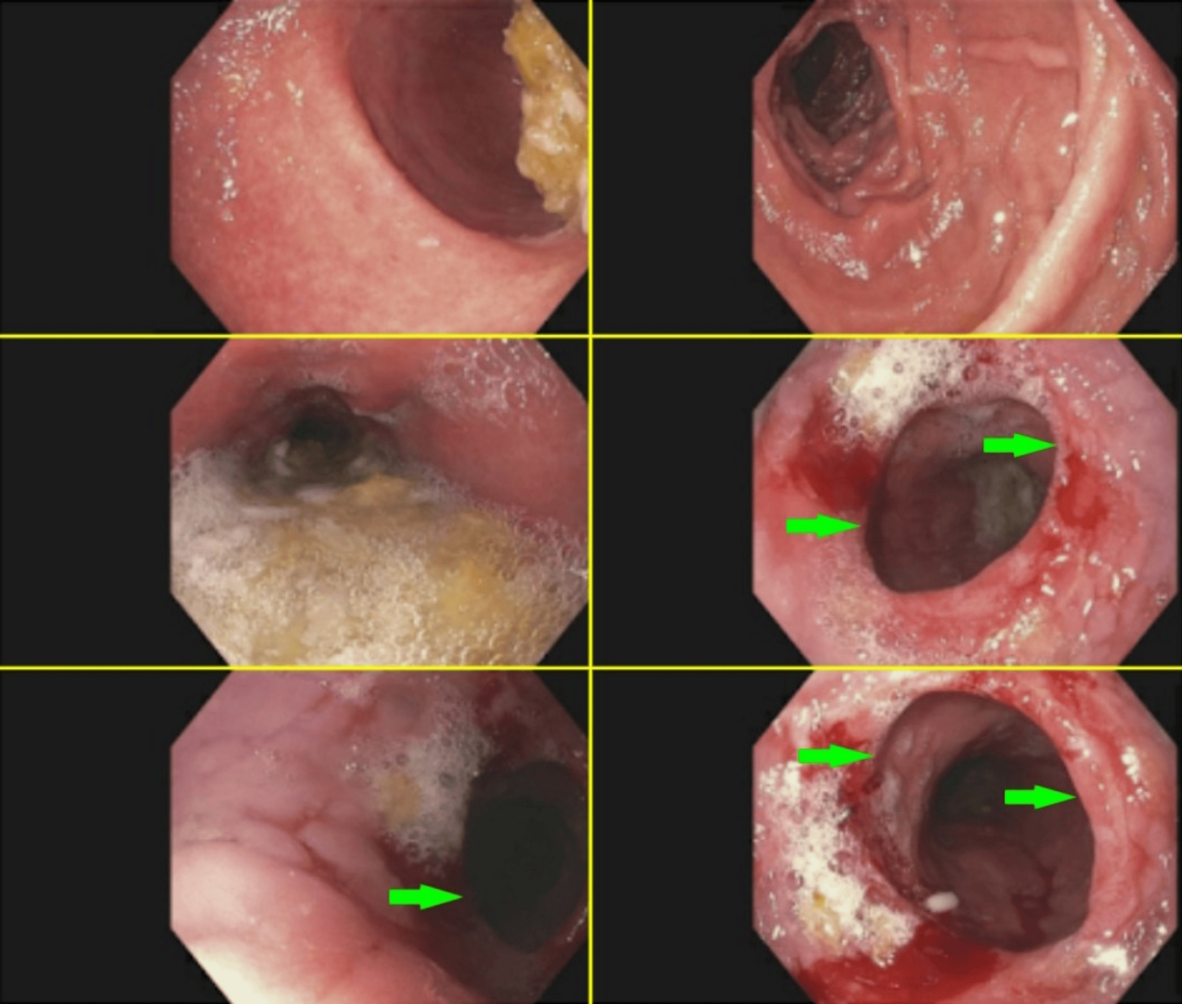
Esophagogastroduodenoscopy with dilation (May 10, 2023) The green arrows point to the same stricture visualized from different angles in the esophagus

In Figure 1, the healed esophagectomy site can be seen from an esophagogastroduodenoscopy with dilation attained five months postoperatively (November 11, 2020). This image is the site of the removed adenocarcinoma 25 cm past the incisors (front teeth), which places the postoperative site at the upper to middle thoracic esophagus. Figure 2 shows images from an esophagogastroduodenoscopy with dilation completed three years postoperatively (May 10, 2023) revealing stricture (red arrows) without evidence of malignant disease recurrence.

As of four years following treatment, the patient is in remission for his esophageal adenocarcinoma, and his cough and dysphagia have since resolved. A CT of the chest, abdomen, and pelvis attained on March 5, 2024, showed esophagectomy without local recurrent or metastatic disease.

## Discussion

This case of esophageal adenocarcinoma was unique in several ways, both in presenting symptoms and lack thereof. This patient required complex multispecialty care needs that crossed state lines, additionally complicating his care. Unusually, this patient’s chief complaint was a chronic cough that was present for over a year. When considering a differential for chronic cough, the most typical etiologies consist of upper airway cough syndrome (postnasal drip), asthma, gastroesophageal reflux disease, and nonasthmatic eosinophilic bronchitis [7]. It was this unusual presentation that led to this patient’s initial assessment via otolaryngology for allergic rhinitis and later evaluation with gastroenterology for gastroesophageal reflux disease. This case of chronic cough highlights the need to consider esophageal pathologies in addition to pulmonary causes, even in cases where respiratory symptoms dominate.

Another unique aspect of this case is the lack of typical symptoms associated with esophageal carcinoma, such as dysphagia, weight loss, or retrosternal pain [8,9]. One such typical case of esophageal adenocarcinoma was reported in a 70-year-old female in 2024 who presented with symptoms of odynophagia, reflux, unintentional weight loss, substernal pain, and abdominal discomfort [10]. Our case presented with a chronic cough and intermittent shortness of breath; however, the patient did develop dysphagia later in the disease course. In the literature, it is highly unusual for a chronic cough to be the initial presenting symptom without the aforementioned typical associated symptoms. One similar case of esophageal adenocarcinoma reported in 2016 was similar in the fact that her chief complaint was a chronic cough over one-year duration without initial dysphagia, weight loss, or retrosternal pain [11]. Unfortunately, this patient elected not to pursue treatment and died nine months after her diagnosis.

This case also illustrates the potential of anchoring bias, concurrent comorbidities complicating care, and the importance of keeping differentials broad despite common etiologies being more likely. The geographic variation in this patient’s history likely contributed to the initial diagnosis of allergic or environmental allergy (i.e., allergic rhinitis) early in the disease course as the patient migrated between two different regions with different climates (Utah to Arizona), which may have delayed gastroenterology referral. This case underscores the importance of equal consideration among secondary and tertiary diagnoses on the differential when treatment for the primary diagnosis fails to alleviate symptoms.

The patient luckily proceeded with the diagnostic test of choice for esophageal carcinoma, which is esophagogastroduodenoscopy with biopsy [12]. This is typically followed by computed tomography and esophageal ultrasound for staging and accurate visualization of the mass’ penetration beyond the esophagus and potential invasion of surrounding organs (lungs, heart, lymph nodes, trachea, aorta, etc.). This patient was deemed a good candidate for surgical excision of the mass following chemoradiation and was in remission four years after surgical removal. This case demonstrates a successful outcome following comprehensive multidisciplinary intervention. This patient received workup and treatment from cardiology, allergy, gastroenterology, and oncology, leading to a successful response to chemoradiation and esophagectomy. This highlights the lesson that complex cases benefit from timely interdisciplinary collaboration to improve outcomes in atypical presentations.

## Conclusions

In this case report, we discussed the unique presentation of chronic cough as the chief complaint of a patient who was eventually found to have esophageal adenocarcinoma. Given the limited reports on such cases, further insight may be required regarding the initial presentation of esophageal carcinoma to better guide physicians when creating their differential diagnoses. Red flags to look for that this case sheds light on include a worsening of symptoms despite appropriate treatment for the primary suspects of chronic cough. This is especially important if the patient develops GI symptoms (such as dysphasia in this case) and if treatment for suspected gastroesophageal reflux disease with the recommended trial of proton pump inhibitor medication does not abate or even improve the patient's symptoms. Emphasizing higher suspicion in cases of chronic cough that do not resolve with first-line treatments for the typically suspected etiologies (upper airway cough syndrome formerly known as postnasal drip, asthma, gastroesophageal reflux disease, nonasthmatic eosinophilic bronchitis) is essential for faster detection and earlier treatment of such cases of esophageal carcinoma.
